# Characteristics of the Built Environment in Relation to Objectively Measured Physical Activity Among Mexican Adults, 2011

**DOI:** 10.5888/pcd11.140047

**Published:** 2014-08-28

**Authors:** Deborah Salvo, Rodrigo S. Reis, Areyh D. Stein, Juan Rivera, Reynaldo Martorell, Michael Pratt

**Affiliations:** Author Affiliations: Rodrigo S. Reis, Pontificia Universidade Catolica do Parana, Curitiba, Brazil, and Federal University of Parana, Curitiba, Brazil; Areyh D. Stein, Reynaldo Martorell, Emory University, Atlanta, Georgia; Juan Rivera, Nutrition and Health Research Center, National Institute of Public Health, Cuernavaca, Mexico; Michael Pratt, Emory University, Atlanta, Georgia, and Centers for Disease Control and Prevention, Atlanta, Georgia. Dr Salvo is also affiliated with the Stanford Prevention Research Center, Stanford University School of Medicine, Stanford, California. At the time of the research, she was a doctoral student at Emory University, Atlanta, Georgia.

## Abstract

**Introduction:**

The built environment correlates of physical activity are documented in high-income countries but have yet to be studied among Mexican adults. Our objectives were to assess the associations between characteristics of the built environment and physical activity among adults in Cuernavaca, Mexico, and to examine potential moderation by perceived park and neighborhood safety.

**Methods:**

We conducted a population-based study of adults in Cuernavaca, Mexico, in 2011 (N = 677). Participants wore Actigraph GT3X accelerometers for 7 days. We used geographic information systems (GIS) to generate 500-m- and 1-km-buffer–based measures of net residential density, proportion of commercial land use, land-use mix, connectivity, walkability, and number of parks and transit routes. We also obtained data on distance to the nearest park with GIS. Perceived neighborhood and park safety were self-reported. We created quartile-based categories for all built environment characteristics and ran linear regression models to estimate the association between each characteristic and total weekly moderate-to-vigorous physical activity (MVPA) and MVPA within 10-minute bouts.

**Results:**

Walkability was inversely related to total weekly minutes of MVPA (1-km buffer, −46.9 [standard error, 20.0]; *P* = .03) and weekly minutes of MVPA within bouts (500-m buffer, −31.5 [12.9]; *P* = .02). The number of transit routes in the 500-m buffer was inversely related to total weekly minutes of MVPA (−23.8 [10.6]; *P* = .04). Perception of park safety moderated the association between physical activity and having a park intersect the 500-m buffer.

**Conclusion:**

Our findings contrast with those from high-income countries, suggesting that environmental programs and policies to increase physical activity in Mexican cities cannot be adapted from high-income countries without considering the local context.

## Introduction

Physical inactivity contributes to 5.3 million annual deaths worldwide and is a risk factor for obesity, diabetes, cardiovascular disease, and cancer (1). Evidence links the built environment and physical activity (2). Walkability (an index incorporating residential density, retail area-to-land ratio, connectivity [ie, number of 3- and 4-way intersections], and land-use mix) is positively associated with physical activity (3), but most evidence is from high-income countries (2). Correlate studies from low-to-middle income countries have recently emerged, and initial findings suggest differences from findings for high-income countries (4,5).

Studies that rely on self-report (as most studies do [2]) are valuable in identifying domains of activity (eg, leisure vs transportation) and environmental perceptions, but objective measures provide more credible evidence for both research and policy (2,6). New technologies, such as accelerometry and geographic information systems (GIS), allow for a more precise estimation of physical activity and the built environment (2,7).

Mexico has an epidemic of obesity and chronic disease (8). The 2012 National Health and Nutrition Survey reported that 71.2% of Mexican adults are overweight or obese, and diabetes and cardiovascular disease are the leading causes of death (8,9). In 2004, physical inactivity accounted for an estimated 4.4% of deaths and 1.2% of disability-adjusted life years among Mexicans (10). However, only 17.2% of adults reported being inactive in 2012 (8). The built environment correlates of physical activity in Mexico are unexplored.

Designing and implementing environmental strategies to increase physical activity requires context-specific studies (2,6,11). Crime and safety may influence physical activity, but studies report inconsistent results (2). Given the current high crime rates in Mexico (9), safety perception may moderate the association of physical activity with objectively measured environmental variables.

The objective of this study was to identify associations between objectively measured physical activity and objectively measured aspects of the built environment among adults in Cuernavaca, Mexico. We hypothesized that the US-based walkability index would not be positively associated with moderate-to-vigorous physical activity (MVPA) in Cuernavaca and that the associations between the built environment and physical activity would be moderated by safety perception.

## Methods

### Study design and site

This was a population-based, multistage cluster study in Cuernavaca, a city of 365,000 inhabitants in central Mexico. Although it is wealthier than the average Mexican city (Human Development Index of 0.86 vs 0.78 for Mexico [12]), Cuernavaca has the socioeconomic, structural, political, and cultural characteristics typical of a low-to-middle income city (12). Crime in Cuernavaca increased during the past decade; homicides increased by 277% (9,13). This study is part of the International Physical Activity and the Environment Network (IPEN)–Mexico project (6).

### Sampling

We collected data in 2011. A representative sample was identified using census tracts as the primary sampling units, which were then stratified into 4 levels of socioeconomic status (SES) based on quartiles and 2 levels of walkability (3) stratified by the median. Eight census tracts per stratum were randomly selected, yielding 32 (of 123 in Cuernavaca) study census tracts. Seven blocks per census tract were randomly selected, and 2 to 4 households per block were randomly selected. Blocks immediately proximal to a census tract with a different SES–walkability stratum were excluded to avoid bias. When a household declined to participate or there was no eligible participant, the household immediately to the right was selected.

Trained field workers recruited 1 participant per household during a home visit. Eligible participants were adults aged 20 to 65 who lived permanently in the household for at least 6 months and had no disability that precluded walking. Participants provided written informed consent; the study was approved by the institutional review boards of Emory University and the Mexican National Institute of Public Health.

### Physical activity measurement and outcomes

Physical activity was measured with ActiGraph GT3X accelerometers (ActiGraph, LLC) using 60-second epochs (counts per minute). Participants wore an accelerometer on their right hip for 7 days at all times except when sleeping, showering, or swimming. Verbal (in person and by demonstration) and written instructions on how to wear the accelerometer were provided. To further ensure protocol compliance, we made 2 telephone calls during the week, and participants indicated their start- and end-time of use per day in an accelerometer log. After 7 days, we made a second home visit to verify wear time. Nonwear time was defined as 60 or more consecutive zeros (1 hour). A valid wear-day was defined as 10 or more valid hours. We validated wear time using MeterPlus 4.2 software (Santech, Inc) and comparing data from the accelerometer log and the telephone calls. Additional information was requested on site from the participant if unusual patterns of daily wear time were observed. If fewer than 5 valid days were recorded, the participant was asked to wear the device for additional days, and a third home visit was scheduled. Days on which we delivered and recovered the accelerometer were considered non-valid. Data were scored with MeterPlus 4.2 using Freedson’s cut-points for adults (14).

We calculated minutes per week of total MVPA and minutes per week of MVPA within 10-minute bouts (Appendix). Bouts were defined as having a minimum duration of 10 minutes, with at least 80% of the bout corresponding to MVPA (ie, all breaks totaled <20% of the bout). If a single break lasted more than 2 minutes, the bout was considered to have been interrupted. A similar definition has been reported (15). Data on bouts were generated in MatLab 7.7 (The MathWorks Inc).

### Objective measurement of the built environment

We assessed the built environment using GIS-derived attributes. The location of each participant’s residence was geocoded using ArcGIS 9.3 (ESRI Inc). One kilometer and 500-m street-network buffers were generated around each participant’s household. Although other studies used a 1-km buffer (16), the greater density and urbanization in some Latin American cities compared with cities in high-income countries (17) suggest that certain features of a built environment in a smaller buffer (500 m) may be more relevant to our study.

We generated the following built environment variables: net residential density, proportion of commercial land use, connectivity (intersection density), land-use mix, walkability index (3), number of parks intersecting the buffer, and number of public transit (bus) routes intersecting the buffer. Because no bus route intersected any 1-km buffer without also intersecting the corresponding 500-m buffer, we used a single variable. We measured the distance to the nearest park by using the street network. We categorized each variable according to city-wide tertiles (for the number of parks intersecting buffer) or quartiles (for all other built environment variables). The category with the smallest value was used as the reference value for each variable (Appendix). GIS data were provided by the Mexican National Institute of Statistics and Geography and the Land Use Registry Department of Cuernavaca. We generated all GIS variables through ArcGIS 9.3.

### Perception of safety

Trained field workers administered the Neighborhood Environment Walkability Scale–Abbreviated (NEWS-A) (18) during the second home visit. Two variables for perceived safety were generated: perception of neighborhood safety and perception of park safety. Both variables were dichotomized as safe or unsafe (Appendix).

### Covariates

Age, sex, education, marital status, individual-level SES (based on 25 questions on household characteristics and assets used by the National Health and Nutrition Survey of Mexico [8]), and motor-vehicle ownership were ascertained by questionnaire during the second home visit. Body mass index (BMI) was measured using Tanita scales and fixed stadiometers (Tanita Corporation of America, Inc), following standardized procedures (19).

### Statistical analysis

All analyses accounted for the multistage clustered design and were weighted for probability of selection and nonresponse by sex, using the survey procedures of SAS 9.3 (SAS Institute Inc). Unadjusted linear regression models for total MVPA and bouts of MVPA were run using each GIS and safety perception variable as independent variables. We ran models adjusting for all covariates. We used SAS’s SURVEYREG procedure, which uses a design-based approach instead of a model-based approach, therefore allowing for linear modeling of non-normally distributed outcomes (20). Regression coefficients are represented as weekly minutes (standard error [SE]) of total MVPA or bouts of MVPA. We used likelihood ratio tests to identify potential interactions between the built environment and perceived safety. If an interaction was significant at *P* < .05, linear regression models were run to estimate the association of the built environment variable with each physical activity outcome in each stratum of perceived safety. The built environment characteristics were also modeled as continuous variables. Statistical significance for all regression analyses, likelihood ratio tests, and tests for linear trend was defined as *P* < .05.

## Results

The study response rate was 58.9%, calculated on the basis of the number of selected households that had an eligible adult. Eight participants were excluded because of invalid accelerometry data, and 7 were excluded because of geocoding problems; the final analytic sample was 662 participants. We found no significant differences in sociodemographic characteristics between the final sample and the excluded participants. The mean age of the sample was 42.0 years; 48.1% were male, 32.7% had education beyond high school, 55.8% were motor vehicle owners, and 31.8% were obese (Table 1); 41.3% perceived their neighborhood as unsafe, and 39.9% perceived their parks as unsafe (Table 2). Participants engaged in MVPA an average 221.3 (standard deviation [SD], 10.1) minutes per week and 63.4 (SD, 4.3) minutes per week within bouts of MVPA; 58.5% met the 150 minutes per week of physical activity recommended by the World Health Organization (21) when considering total MVPA, but 13.3% met the requirement when considering bouts of MVPA.

**Table 1:** Characteristics of Study Participants, Cuernavaca, Mexico, 2011

Sociodemographic variables	n (%^a^) (N = 662) **b**
**Male**	297 (48.1)
**Age, y**	
≤35	217 (33.4)
>35 to ≤50	260 (40.0)
>50 to ≤65	185 (26.6)
**Socioeconomic status^c^**	
Low	196 (31.8)
Medium	163 (24.0)
Medium-high	192 (28.0)
High	111 (16.2)
**Education**	
More than high school	216 (32.7)
**Marital status**	
Single	162 (25.0)
Married^d^	434 (65.3)
Divorced^e^	66 (9.7)
**Motor vehicle ownership**	370 (55.8)
**Body mass index (kg/m^2^)**	
Overweight (25 to <30)	275 (41.2)
Obese (≥30)	210 (31.8)

a Percentages are weighted for probability of selection and nonresponse by sex.

b Final sample (N = 662) excluded 15 participants for whom accelerometry or GIS data were not available; no differences in sociodemographic characteristics were found between the final sample and excluded participants.

c Classifications based on quartiles of index of socioeconomic status; index based on household characteristics and assets.

d Includes living with someone.

e Includes separated and widows.

**Table 2:** Characteristics of the Built Environment and Perceptions of Safety Among Adults, Overall and by Buffer, Cuernavaca, Mexico, 2011

Variable	No. of Respondents (% [SE])^a^ (N = 662)^b^	
**Distance to closest park**		
Very close (<313 m)	165 (16.3 [4.4])	
Medium (313 to <771 m)	166 (25.6 [5.0])	
Far (771 to <1,357 m)	166 (30.9 [5.4])	
Very far (≥1,357 m)	165 (27.2 [6.6])	
**No. of public bus routes per buffer**		
0	81 (11.0 [4.9])	
1 or 2	228 (35.8 [6.9])	
3–7	172 (24.4 [6.3])	
≥8	181 (28.8 [7.1])	
**Neighborhood safety perception (score range, 0–5)**		
Safe (score <3)	387 (58.7 [2.8])	
Unsafe (score ≥3)	275 (41.3 [2.8])	
**Park safety perception (score range, 0–5)**		
Safe (score <3)	382 (60.1 [2.9])	
Unsafe (≥3)	280 (39.9 [2.9])	

**Variable**	**By Buffer^c^**	
	**500 m**	**1 km**
	**n (% [SE])**	**n (% (SE])**

**Net residential density (no. of single family units/km^2^ of residential land use in buffer)**		
Low (<1,583)	190 (27.9 [6.6])	165 (23.6 [4.4])
Medium (1,583 to <2,174)	115 (20.6 [4.4])	173 (30.3 [5.9])
Medium-high (2,174 to <2,730)	115 (20.6 [5.2])	166 (25.0 [4.4])
High (≥2,730)	242 (31.0 [5.7])	158 (21.1 [4.5])
**Proportion of commercial land use (km^2^ designated to commercial land use/km^2^ of buffer)**		
Low (0%)	249 (37.8 [6.4])	154 (25.0 [5.4])
Medium (0% to <15%)	119 (18.1 [4.8])	183 (27.5 [4.7])
Medium-high (15% to <25%)	132 (22.5 [4.4])	162 (26.4 [4.2])
High (≥25%)	162 (21.6 [4.4])	163 (21.1 [4.6])
**Land-use mix^d^> **		
Low (<0.25)	224 (34.4 [5.9])	135 (21.7 [5.9])
Medium (0.25 to <0.40)	208 (31.7 [4.1])	224 (29.1 [4.6])
Medium-high (0.40 to <0.50)	135 (17.8 [3.9])	169 (27.2 [5.3])
High (≥0.50)	95 (16.0 [4.7])	134 (22.0 [5.0])
**Connectivity (no. of 3- and 4-way intersections/km^2^ of buffer)**		
Low (<111)	166 (25.7 [4.2])	166 (25.7 [5.6])
Medium (111 to <135)	167 (26.2 [3.7])	161 (22.1 [3.9])
Medium-high (135 to <167)	166 (26.0 [2.8])	166 (25.8 [7.0])
High (≥167)	163 (22.1 [6.1])	169 (26.4 [5.8])
**Walkability index^d^**		
Low (less than −45)	148 (25.4 [6.6])	155 (23.3 [6.5])
Medium (−45 to <15)	231 (33.4 [4.3])	174 (30.3 [5.1])
Medium-high (15 to <50)	135 (20.4 [3.7])	187 (27.3 [4.0])
High (≥50)	148 (20.8 [4.2])	146 (19.1 [4.9])
**No. of parks per buffer**		
0	415 (72.9 [7.7])	236 (40.9 [6.5])
1	165 (20.9 [6.8])	231 (36.5 [5.9])
≥2	82 (6.3 [5.8])	195 (22.5 [6.3])

Abbreviations: SE, standard error.

a Percentages were weighted for probability of selection and nonresponse by sex.

b Final sample (N = 662) excluded 15 participants for whom accelerometry or GIS data were not available; no differences in sociodemographic characteristics were found between the final sample and excluded participants.

c For these variables, 1-km and 500-m street-network buffers were generated around each participant’s household.

d See Appendix for definition.

Having 8 or more bus routes intersecting the 500-m buffer was inversely related to total MVPA (−23.8 [0.6] min; *P* = .04) (Table 3) but not to MVPA within bouts (−7.0 [12.3] min; *P* = .58) (Table 4). We found no significant linear trend for the relationship between the number of transit routes and total MVPA or MVPA within bouts; neither did we find any significant association between distance to the closest park and total MVPA or MVPA within bouts.

**Table 3:** Association of Total Minutes Per Week of Moderate-to-Vigorous Physical Activity With Environmental Variables Among Mexican Adults, Overall and by Buffer, Cuernavaca, Mexico, 2011

Variable	Regression Estimate (SE) [*P* Value]			
	Unadjusted		Adjusted^a^	
**Neighborhood safety**				
Safe	0		0	
Unsafe	0.23 (16.38) [.99]		−1.33 (18.50) [.94]	
**Park safety**				
Safe	0		0	
Unsafe	−17.19 (10.11) [.11]		−23.17 (9.16) [.08]	
**Distance to park**				
Very close (<313 m)	0		0	
Medium (313 to <771 m)	−2.43 (16.86) [.87]		12.93 (16.18) [.43]	
Far (771 to <1,357 m)	−4.81 (22.20) [.83]		6.13 (16.61) [.72]	
Very far (≥1,357 m)	10.59 (24.19) [.67]		15.56 (18.02) [.40]	
**No. of public bus routes per buffer**				
0	0		0	
1	−12.69 (19.50) [.52]		−7.76 (20.02) [.70]	
2–7	−17.99 (19.43) [.36]		−15.55 (23.82) [.52]	
≥8	−51.72 (15.62) [.002]		−23.78 (10.61) [.04]	

**Variable**	**By Buffer^b^**			
	**500 m**		**1 km**	
	**Unadjusted**	**Adjusted^a^**	**Unadjusted**	**Adjusted^a^**

**Net residential density (no. of single family units/km^2^ of residential land use in buffer)**				
Low (<1,583)	0	0	0	0
Medium (1,583 to <2,174)	1.02 (27.01) [.87]	−4.72 (23.1) [.84]	−37.60 (17.02) [.04]	−39.88 (18.20) [.04]
Medium-high (2,174 to <2,730)	−9.75 (26.88) [.72]	−39.10 (22.65) [.16]	−30.38 (21.45) [.24]	−40.42 (22.61) [.08]
High (≥2,730)	−16.12(23.56) [.50]	−30.58 (18.69) [.20]	6.20 (12.31) [.81]	−15.99 (18.14) [.49]
**Proportion of commercial land use (km^2^ designated to commercial land use/km^2^ of buffer)**				
Low (0%)	0	0	0	0
Medium (0% to <15%)	0.67 (21.47) [.98]	−2.77 (20.55) [.89]	−9.00 (25.80) [.73]	−0.37 (24.17) [.99]
Medium-high (15% to <25%)	−7.88 (24.93) [.75]	−9.48 (21.06) [.66]	−44.31 (20.64) [.04]	−26.26 (20.43) [.21]
High (≥25%)	−73.18 (16.92) [<.001]	−54.51 (15.53) [<.001]^c^	−35.53 (22.13) [.12]	−20.27 (20.85) [.34]
**Land-use mix^d^**				
Low (<0.25)	0	0	0	0
Medium (0.25 to <0.40)	11.03 (18.18) [.55]	6.29 (18.55) [.74]	10.92 (19.07) [.57]	14.44 (19.32) [.46]
Medium-high (0.40 to <0.50)	−36.34 (24.06) [.14]	−25.61 (20.89) [.23]	−18.09 (15.34) [.25]	−11.20 (15.24) [.47]
High (≥0.50)	−4.33 (26.73) [.87]	−12.68 (19.76) [.53]	−1.14 (24.20) [.96]	−0.39 (21.75) [.99]
**Connectivity (no. of 3- and 4-way intersections/km^2^ of buffer)**				
Low (<111)	0	0	0	0
Medium (111 to <135)	−31.87 (29.88) [.30]	−36.14 (22.05) [.11]	−6.93 (21.90) [.75]	−21.55 (16.94) [.21]
Medium-high (135 to <167)	−16.58 (30.20) [.59]	−22.63 (31.40) [.48]	−41.20 (18.82) [.04]	−35.51 (14.67) [.02]
High (≥167)	−3.71 (18.49) [.84]	−18.68 (18.25) [.32]	−16.71 (20.40) [.42]	−32.07 (17.49) [.09]
**Walkability index^d^**				
Low (less than −45)	0	0	0	0
Medium (−45 to <15)	−23.78 (22.19) [.29]	−24.35 (18.31) [.20]	−8.35 (18.02) [.65]	−20.42 (18.91) [.29]
Medium-high (15 to <50)	−19.92 (30.83) [.52]	−27.11 (26.53) [.32]	−36.07 (21.03) [.10]	−12.89 (21.54) [.55]
High (≥50)	−25.88 (26.16) [.33]	−34.30 (21.69) [.13]^c^	2.59 (23.42) [.91]	−46.91 (20.04) [.03]
**No. of parks**				
0	0	0	0	0
1	−13.23 (14.96) [.38]	−27.87 (14.90) [.05]	−19.25 (19.03) [.32]	−10.82 (16.98) [.53]
>2	45.03 (43.78) [.31]	31.61 (35.60) [.38]	5.25 (18.58) [.78]	−3.27 (17.73) [.86]

a Adjusted models control for total accelerometer wear time, sex, age, individual socioeconomic status, education, marital status, motor vehicle ownership, and body mass index.

b For these variables, 1-km and 500-m street-network buffers were generated around each participant’s household.

c Significant linear trend (*P* < .05).

d See Appendix for definition.

**Table 4:** Association of Minutes Per Week of 10-Minute Bouts^a^ of Moderate-to-Vigorous Physical Activity With Environmental Variables Among Mexican Adults, Overall and by Buffer, Cuernavaca, Mexico, 2011

Variable	Regression Estimate (SE) [*P* Value]			
	Unadjusted		Adjusted^b^	
**Neighborbood safety**				
Safe	0		0	
Unsafe	−5.00 (6.55) [.45]		−2.61 (7.38) [.73]	
**Park safety**				
Safe	0		0	
Unsafe	−10.60 (6.56) [.09]		−12.00 (7.01) [.05]	
**Distance to park**				
Very close (<313 m)	0		0	
Medium (313 to <771 m)	5.17 (6.76) [.45]		9.91 (7.00) [.17]	
Far (771 to <1,357 m)	4.92 (8.68) [.58]		8.16 (7.72) [.30]	
Very far (≥1,357 m)	4.44 (10.18) [.67]		8.24 (11.51) [.48]	
**No. of public bus routes per buffer**				
0	0		0	
1	2.66 (0.26) [.80]		−1.59 (11.09) [.89]	
2–7	−5.39 (8.13) [.51]		−4.51 (11.42) [.70]	
≥8	−10.70 (11.29) [.35]		−6.95 (12.29) [.58]	

**Variable**	**By Buffer^c^**			
	**500 m**		**1 km**	
	**Unadjusted**	**Adjusted^b^**	**Unadjusted**	**Adjusted^b^**

**Net residential density (no. of single family units/km^2^ of residential land use in buffer)**				
Low (<1,583)	0	0	0	0
Medium (1,583 to <2,174)	−3.87(11.59) [.74]	−7.97 (12.00) [.51]	−21.28 (7.12) [.04]	−24.32 (6.24) [.03]
Medium-high (2,174 to <2,730)	−15.87 (7.72) [.17]	−22.87 (10.55) [.05]	−16.95 (12.80) [.15]	−23.34 (10.88) [.05]
High (≥2,730)	−10.46 (9.74) [.40]	−21.68 (9.32) [.04]	−1.55 (15.22) [.91]	−8.92 (10.30) [.50]
**Proportion of commercial land use (km^2^ designated to commercial land use/km^2^ of buffer)**				
Low (0%)	0	0	0	0
Medium (0% to <15%)	3.20 (10.70 [.77]	−0.82 (10.50) [.94]	−0.91 (9.92) [.93]	1.56 (9.75) [.87]
Medium-high (15% to <25%)	−4.12 (9.07) [.65]	−8.59 (9.48) [.37]	−7.46 (11.07) [.51]	−5.70 (10.84) [.60]
High (≥25%)	−32.64 (8.32) [<.001]^d^	−33.96 (8.28) [.001]^d^	−16.35 (10.79) [.14]	−20.31 (13.42) [.11]
**Land-use mix^d^**				
Low (<0.25)	0	0	0	0
Medium (0.25 to <0.40)	0.73 (8.60) [.93]	−5.49 (9.06) [.55]	2.93 (10.34) [.78]	1.03 (11.17) [.93]
Medium-high (0.40 to <0.50)	−19.58 (14.46) [.19]	−20.65 (12.96) [.12]	−20.72 (8.57) [.02]	−22.33 (9.64) [.03]
High (≥0.50)	−16.44 (11.62) [.17]	−19.17 (11.63) [.11]	−12.42 (13.10) [.35]	−17.34 (8.27) [.05]
**Connectivity (number of 3- and 4-way intersections/km^2^ of buffer)**				
Low (<111)	0	0	0	0.
Medium (111 to <135)	−8.79 (11.98) [.47]	−8.68 (10.15) [.40]	−6.58 (10.32) [.53]	−9.24 (10.63) [.39]
Medium-high (135 to <167)	−8.33 (17.49) [.64]	−11.54 (16.65) [.49]	−13.75 (9.10) [.14]	−18.33 (10.37) [.09]
High (≥167)	−7.95 (9.93) [.43]	−10.35 (10.63) [.34]	−5.95 (9.92) [.55]	−9.48 (6.91) [.12]
**Walkability index^d^**				
Low (less than −45)	0	0	0	0
Medium (−45 to <15)	−9.84 (12.18) [.43]	−10.22 (11.47) [.38]	−16.79 (9.48) [.09]	−22.25 (10.22) [.04]
Medium-high (15 to <50)	−16.54 (12.23) [.19]	−18.31 (12.94) [.17]	−28.23 (9.31) [.01]	−34.21 (10.01) [<.001]
High (≥50)	−29.89 (11.96) [.02]^d^	−31.49 (12.93) [.02]^d^	−6.25 (12.47) [.62]	−12.65 (13.19) [.35]
**No. of parks**				
0	0	0	0	0
1	−11.96 (6.24) [.07]	−16.84 (8.19) [.03]	−0.02 (11.80) [.99]	0.24 (12.38) [.98]
≥2	25.91 (27.18) [.35]	20.51 (26.16) [.44]	1.15 (10.44) [.91]	−2.17 (11.13) [.85]

a Only activity recorded as moderate-to-vigorous bouts of at least 10 minutes; at least 80% of bout had to be moderate-to-vigorous activity (ie, no breaks of ≥2 minutes).

b Adjusted models control for total accelerometer wear time, sex, age, individual socioeconomic status, education, marital status, motor vehicle ownership, and BMI status.

c For these variables, 1-km and 500-m street-network buffers were generated around each participant’s household.

d Significant linear trend (*P* < .05).

Participants who had 1 park intersecting the 500-m buffer engaged in 27.9 (14.9) fewer minutes per week of total MVPA (*P* = .05) and 16.8 (8.2) fewer minutes of MVPA within bouts (*P* = .03) than participants with no parks intersecting the 500-m buffer. We found no significant association for participants with 2 or more intersecting parks for total MVPA or MVPA within bouts (using 0 parks as reference), and no significant linear trends were found for this relationship. 

The walkability index in the 1-km buffer was inversely associated with total MVPA (−46.9 [20.0] min; *P* = .03 for highest vs lowest quartile of walkability). For the 500-m buffer, a significant inverse linear trend (*P* = .02) was identified for the association of walkability with total MVPA. High walkability in the 500-m buffer was related to 31.5 (12.9) fewer minutes per week of MVPA within bouts (*P* = .02) when compared with low walkability (quartile 1), and the inverse linear trend between walkability and bouts of MVPA persisted (*P* = .01). For walkability within the 1-km buffer, quartile 2 (medium, −22.3 [10.2] min; *P* = .04) and quartile 3 (medium-high, −34.2 [10.01] min; *P* = <.001) but not quartile 4 were negatively associated with bouts of MVPA, using quartile 1 (low walkability) as the reference. The linear trend was not significant for the relationship between walkability in the 1-km buffer and MVPA within bouts. We found similar significant inverse associations between both physical activity outcome variables (total MVPA and MVPA within bouts) and the individual components of the walkability index as defined for high-income countries (3).

Perceived neighborhood safety was not significantly associated with total MVPA or MVPA within bouts. Unsafe park perception was inversely associated with total MVPA (−23.2 [9.2] min; *P* = .08) and MVPA within bouts (−12.0 [7.0] min; *P* = .05). The association between number of parks intersecting the 500-m buffer with both total MVPA and MVPA within bouts was moderated by perceived park safety (tests for interaction, total MVPA, *P* = .04; bouts of MVPA, *P* = .02). When parks were perceived as unsafe, having 1 park intersect the 500-m buffer was associated with 30.8 (14.9) (*P* = .047) and 19.2 (7.2) (*P* = .03) fewer weekly minutes of total MVPA and bouts of MVPA respectively, whereas no significant association was found when parks were perceived as safe.

## Discussion

The associations of objectively measured physical activity with objectively measured aspects of the built environment for a representative sample of adults in Cuernavaca differ markedly from associations reported for high-income countries. Our results suggest that the relationship between the built environment and physical activity may be context-specific, and that the context in Mexico differs markedly from that in high-income countries.

In contrast to reports from high-income countries (22), the number of bus routes in our study was inversely associated with physical activity. The exact location of bus stops is not provided by transportation authorities, which is characteristic of public transit in Mexico, where it is common practice to signal a bus to stop anywhere along its route. Our results are consistent with a study in Bogotá, Colombia, which has a similar transit system (5). Positive associations with physical activity were found for proximity to bus stops more than 500 meters apart in Bogotá (23), suggesting that if norms for use of designated bus stops changed, physical activity among users might increase. This is relevant for Cuernavaca because about half of adults do not own motor vehicles.

Despite high crime rates in Cuernavaca (9,13), neighborhood safety perception was not associated with physical activity, nor did it moderate any of the relationships with built environment variables. However, park safety perception elucidated the negative association between having 1 park in the 500-m buffer and having lower levels of weekly physical activity. It was only when parks were perceived as unsafe that the presence of a park in the 500-m buffer was inversely related to physical activity; no association was found when parks were perceived as safe.

Our findings on the walkability index (3) have research and public health implications for low-to-middle income countries. Studies from the United States, Europe, and Australia showed positive associations between physical activity and intersection density (connectivity), land-use mix, and residential density (3,24–26). These elements were combined into a walkability index (3). Our results show an inverse association between this index (or its individual components) and physical activity.

Although findings from Curitiba, Brazil, show associations between physical activity and walkability consistent with those from high-income countries (27), in Bogotá, Colombia, researchers using GIS-derived data and self-reported physical activity reported no associations between residential density or land-use mix and physical activity, which may reflect scale differences because almost all neighborhoods were dense with mixed-use (23,28). Similar results to ours were reported in Bangladesh (29). Studies used sample-specific definitions of high and low walkability, but low density, low connectivity, or low land-use mix in Cuernavaca may be equivalent to high density, high land-use mix, and high connectivity in high-income countries. Perhaps neighborhoods that are too dense, mixed, or connected represent a barrier for physical activity, and the association of physical activity with walkability may be of an inverse *U*-shape rather than linear. Our data are insufficient to test this hypothesis. Analyses of the full IPEN data set could help address this question.

Our results highlight the complexity of the relationships between the built environment and physical activity among Mexican adults. Medium and medium-high but not high net residential density in the 1-km buffer were associated with lower levels of total MVPA and bouts of MVPA when compared with low density, suggesting a threshold for this relationship. We observed similar nonlinear relationships for other built environment characteristics. Meanwhile, inverse linear trends were found for the association of both total MVPA and bouts of MVPA with the US-based walkability index and the proportion of commercial land-use in the 500-m buffer. Some characteristics showed stronger associations with physical activity for the 500-m buffer only (number of parks per buffer, commercial land-use proportion), while for others the 1-km buffer yielded more significant associations (residential density, land-use mix), suggesting that some environmental features are related to physical activity only in a microenvironment. Some features were more strongly associated with bouts of MVPA (residential density, land-use mix, walkability, number of parks) and others with total MVPA (number of transit routes). More studies are needed to elucidate these complex relationships.

Out study has several limitations, including a cross-sectional design, self-reported data, and a low response rate (but consistent with other studies in Mexico [8]). We did not examine the association of park type with physical activity. Objective data on neighborhood crime were not available. Only land-cover (vs parcel-level) data were available for land use, possibly decreasing precision for these variables. GIS pedestrian-enhanced street layers were not available. More accessible GIS data are required to better study built environment associations in Mexico. Objective measures provide excellent estimates of physical activity and the built environment but do not allow the study of physical activity domains and neighborhood perceptions that self-report tools can allow. This shortcoming of objective measures was demonstrated by the significant moderation by park safety perception of the association of number of parks with physical activity. Analyses of transportation and leisure-time physical activity might better elucidate these relationships.

Our study has many strengths. It is the first Latin American study to examine physical activity and the built environment using objective measures for both dependent and independent variables. As part of IPEN, state-of-the-art methods, measures, and instruments were used (6). The use of a non-normalized physical activity outcome variable responded to the need for studies that treat physical activity as a continuous variable (2,21,30). By using quartiles for the environmental variables instead of dichotomizing or assigning *z* scores, we identified more associations and observed complex relationships between physical activity and the built environment. Our study is the first to provide estimates of the built environment correlates of physical activity among urban Mexican adults. Our findings have public health implications for Mexico and potentially other low-to-middle income countries, showing associations of physical activity with walkability that are discordant with those observed in high-income countries (3) and suggesting that caution should be taken when translating evidence from high-income countries to low-to-middle income countries (4,5,11).

## Appendix. Definitions of Physical Activity Outcomes, Environmental Variables, and Covariates

**Table:**

Variable	Type	Definition
**Physical activity outcomes**		
Total minutes of MVPA per week	Continuous	Total minutes per week of activity ≥1,952 counts per minute^a^.
Minutes of MVPA within bouts	Continuous	Minutes per week of activity ≥1,952 counts per minute^a^, recorded within bouts of MVPA as defined for this study
MVPA bout	—	All of the following characteristics were required for a bout of MVPA: 1) duration of at least 10 minutes; 2) intensity of activity is moderate to vigorous (≥1,952 counts per minute); 3) ≥80% of bout consists of moderate-to-vigorous intensity of activity (≥1,952 counts per minute); therefore, ≤20% of the bout could correspond to breaks of ≤1,952 counts per minute; 4) each break of ≤1,952 counts per minute^a^ has a maximum duration of 2 minutes. If requirement no.3 or no. 4 was not met, the bout was considered interrupted.
**Environmental variables**		
Net residential density (500 m and 1 km)	Categorical	Number of single family units per buffer area/total squared kilometers of residential land use in buffer area. Categories^b^ were defined as low (reference), <1,582.99; medium, 1,582.99 to <2,174.19; medium-high, 2,174.19 to <2,729.75; high, ≥2,729.75. These values were rounded to whole numbers for reporting purposes.
Proportion of commercial land use (500 m and 1 km)	Categorical	Squared kilometers designated to commercial land use in buffer area/total squared kilometers of buffer area. Categories^b^ were defined as low (reference); 0%; medium, 0% to less than15%: medium-high, 15% to less than 25%; high, ≥25%.
Connectivity (500 m and 1 km)	Categorical	Intersection density defined as the number of 3- and 4-way intersections in a buffer area/total buffer area in squared kilometers. Categories^b^ were defined as low (reference), <111.03; medium, 111.03 to <135.33; medium-high, 135.33 to <166.59; high: ≥166.59. Values were rounded to whole numbers for reporting purposes.
Land-use mix (500 m and 1 km])	Categorical	−[∑*i* (*pi*)(ln *pi*)]/(ln *k*) where *p* = proportion of total land uses, *i* = land use category, ln = natural logarithm, *k* = number of land uses. Range is 0 to 1. Categories^b^ are defined as low (reference), <0.25; medium, 0.25 to <0.40; Medium-high, 0.40 to <0.50; high, ≥0.50.
Walkability index	Categorical	*z*-scored net residential density + *z*-scored commercial land use proportion + 2(*z*-scored connectivity) + *z*-scored land-use mix^c^. Categories^b^ are defined as low (reference), less than −45; medium, −45 to <15, medium-high, 15 to <50; high, ≥50.
Distance to closest park	Categorical	Distance in meters to the nearest park. Categories^b^ are defined as near (reference), <313.116; medium, 313.116 to <771.156; far, 771.156 to <1,356.51; very far, ≥1,356.51. These values were rounded to whole numbers for reporting purposes.
Number of parks (500 m and 1 km])	Categorical	Number of parks intersecting a buffer (ie, includes parks fully in and parks partially in a buffer). Categories are 0 parks in buffer (reference); 1 park in buffer; ≥2 parks in buffer.
Public transportation routes	Categorical	Number of bus public transit routes intersecting the buffer. No difference was found between 500-m and 1-km buffers (ie, no public transit route intersected the 1-km buffer while not intersecting the 500-m buffer). Therefore, a unique variable was used. Categories were based on quartiles: 0 routes (reference), 1 or 2 routes, 3–7 routes, ≥8 routes.
Neighborhood safety perception	Categorical	Binary: 1 = unsafe neighborhood, 0 = safe neighborhood. Based on average score (score range, 1–4]) of 5 NEWS–A^d^ items (1 = lowest agreement, 4 = highest agreement]): The crime rate in my neighborhood is high, the crime rate in my neighborhood makes it unsafe to walk during the day, the crime rate in my neighborhood makes it unsafe to walk during the night, the parks and plazas in my neighborhood are unsafe to visit during the day, the parks and plazas in my neighborhood are unsafe to visit during the night. A score ≥3 was categorized as unsafe; a score <3, safe (reference).
Park safety perception	Categorical	Binary: 1 = unsafe park, 0 = safe park. Based on average score (score range 1–4) of 2 NEWS–A^d^ items (1 = lowest agreement, 4 = highest agreement]): The parks and plazas in my neighborhood are unsafe to visit during the day, the parks and plazas in my neighborhood are unsafe to visit during the night. A score ≥3 was categorized as unsafe; a score <3, safe (reference).
**Covariates**		
Sex	Categorical	Binary: 1 = male, 0 = female (reference).
Age	Continuous	Range, 20–65 years.
Individual socioeconomic status (SES)	Categorical	Low (reference), medium, medium-high, high. Based on quartiles of individual SES index, constructed using centralized *z* scores from a set of 25 questions on household characteristics and assets per participant. The index excluded motor vehicle ownership and education.
Education level	Categorical	Binary: 0 = high school or less (≤12 y of education) (reference), 1 = more than high school (>12 y of education).
Marital status	Categorical	Single (not living with a partner) (reference); married (includes living with a partner); divorced (includes separated and widows.
Motor vehicle ownership	Categorical	Binary: 1 = yes (owning at least 1 car or motorcycle), 0 = no (reference).
Body mass index (BMI)	Categorical	BMI (kg/m^2^) <25 (normal) (reference); 25 to <30 (overweight); ≥30 (obese).

^a^ Source: Freedson et al (14).

^b^ Defined according to city-wide quartiles using census-tract–level data for Cuernavaca.

^c^ Source: Frank et al (3).

^d^ Neighborhood Environment Walkability Scale–Abbreviated (NEWS-A) (18).
